# Supplementary material for: Water Filtration Membranes Based on Non-Woven Cellulose Fabrics: Effect of Nanopolysaccharide Coatings on Selective Particle Rejection, Antifouling, and Antibacterial Properties
Source: Nanomaterials (Basel). 2021 Jul 5;11(7):1752. doi: 10.3390/nano11071752 (PMC8308125; doi:10.3390/nano11071752)
Supplement: Supplementary file 1 [file nanomaterials-11-01752-s001.zip › nanomaterials-1278049-supplementary.pdf]

nanomaterials

Supplementary Materials

Water Filtration Membranes Based on Non-Woven Cellulose Fabrics: Effect of Nanopolysaccharide Coatings on Selective Particle Rejection, Antifouling, and Antibacterial Properties

Blanca Jalvo, Andrea Aguilar-Sanchez, Maria-Ximena Ruiz-Caldas and Aji P. Mathew *

Department of Materials and Environmental Chemistry, Stockholm University, Frescativägen 8, 10691, Stockholm, Sweden; Blanca.Jalvo@aces.su.se (B.J.); andrea.aguilar@mmk.su.se (A.A.-S.); mariaximena.ruizcaldas@mmk.su.se (M.-X.R.-C.)

* Correspondence: aji.mathew@mmk.su.se; Tel: +46-816-1256

S1. Chitin nanocrystals preparation process.

Chitin nanocrystals (ChNC) were prepared via hydrochloric acid hydrolysis (Goodrich & Winter, 2007; Larbi et al., 2018, Please add Company, City, State if possible (USA and Canada), Country). Briefly, deproteinized and bleached shrimp chitin flakes (Sigma-Aldrich, Please add City, Germany) underwent an acid hydrolysis reaction with 3N hydrochloric acid at 90 °C for 90 min. When the reaction was completed, the resulting suspension was centrifuged to remove the excess of acid and subsequently to collect the turbid supernatant containing the chitin nanocrystals. This collected fraction, the chitin nanocrystal suspension, was dialyzed against distilled water to achieve a 5.6 pH suspension and finally homogenized and sonicated to ensure separation of the individual nanocrystals prior to storage.

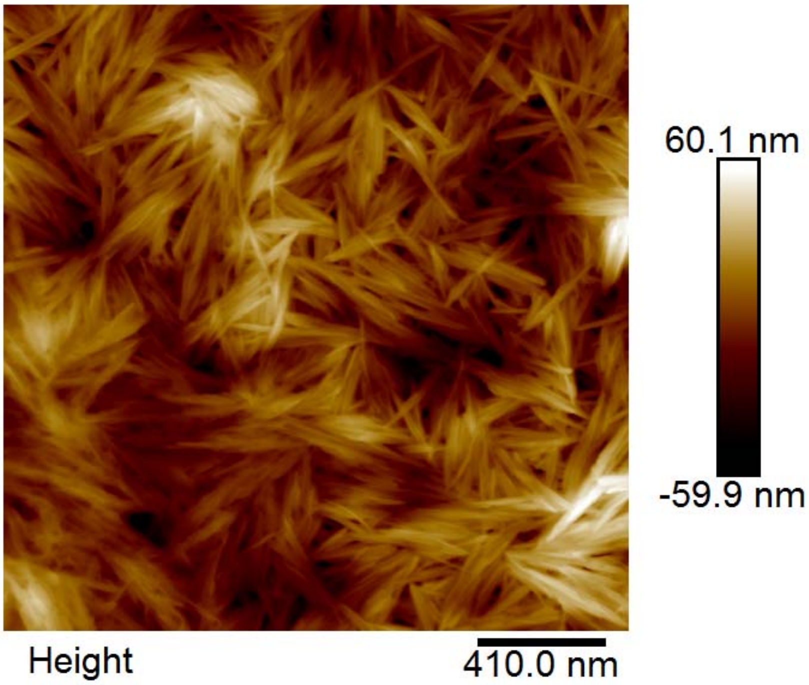

Figure S1. AFM 2D topography of ChNC.

S2. Optimization of the coating process.

The coating process parameters were optimized based on the optimal flux values and the stability of the coating. Variable studied were coating speed within a range of 1–50 mm/s, the gap distance of 50, 100 and 150 μm, and the different polysaccharide suspension concentration of 1 wt%, 3 wt% and 5 wt%. the best membrane performance was obtained when the parameters were of 1 wt% polyssaccharide suspension, 40 mm/s coated speed, and 150 μm wet coating thickness, hence presented in this work.

References

1. Goodrich, J.D., Winter, W.T. α-Chitin nanocrystals prepared from shrimp shells and their specific surface area measurement. *Biomacromolecules*, **2007**, *8(1)*, 252–257.
2. Larbi, F., García, A., del Valle, L.J., Hamou, A., Puiggalí, J., Belgacem, N., Bras, J. Comparison of nanocrystals and nanofibers produced from shrimp shell α-chitin: From energy production to material cytotoxicity and Pickering emulsion properties. *Carbohydrate Polymers*, 2018, *196*, 385–397.
